# Osteo-Cartilaginous Deposit in the Knee Joint

**Published:** 1877-04

**Authors:** Chas. L. Summey

**Affiliations:** Stone Mountain


STONE MOUNTAIN, April 5, 1877.

MESSRS. EDITORS: The following report of a case of osteo-cartilaginous deposit in the knee joint may be of interest to some one of your readers, and so, if you deem it worthy the space in your Journal, you are welcome to it:

M. C., col., æt. 65, came to me February 1, 1876, complaining of his knee hurting him, with inability to walk. Upon examination I found a hard circumscribed tumor situated within the synovial membrane. I could move it from external condyle, under the quadriceps extensor, to the internal condyle. The patient stated that he first noticed it about ten years ago, and that it had been gradually growing since that time. He also stated that he had been a sufferer from rheumatism all his life, up to within a few years since. Upon the danger and necessity of an operation for its removal being explained to him, and his consent being obtained, on Feb. 6, 1876, assisted by Dr. Thomas Wootten, I proceeded to operate.

The tumor being brought around over the internal condyle, was grasped by thumb and finger, and a longitudinal incision two inches long was made into the synovial sac. This was followed by escape of the fluid with the body accompanying it, as it was retained by no attachments. It was found to be in shape superiorly convex, inferiorly concave, one and a half inches long, three quarters wide, and one-half inch thick.

The edges of the wound were brought together and retained by adhesive strips, and the leg placed comfortably on a well padded splint with oilcloth covering; perfect quiet was enjoined. When I saw him again, on the night of the 7th, some tumefaction and a little pain was present. Ordered ice cold applications and perfect quiet, with the occasional administration of an opiate.

The wound healed by the first intention, without much constitutional disturbance; considerable tumefaction with scarcely any pain was, for awhile, noticed. This was probably the result of irritation to the synovial membrane and an increased secretion of synovia. He was kept in bed until all inflammation had subsided, and then instructed to use crutches for a month as a safeguard against any irritating tendency from use of the joint.

I saw him again on March 4, 1877, and fouud him perfectly well, and with complete use of the affected joint.

Very truly, CHAS. L. SUMMEY, M.D.

